# Liver Iron Loading in Alcohol-Associated Liver Disease

**DOI:** 10.1016/j.ajpath.2022.08.010

**Published:** 2022-10-25

**Authors:** Najma Ali, Kevin Ferrao, Kosha J. Mehta

**Affiliations:** ∗ GKT School of Medical Education, Faculty of Life Sciences and Medicine, King's College London, London, United Kingdom; † Centre for Education, Faculty of Life Sciences and Medicine, King's College London, London, United Kingdom

## Abstract

Alcohol-associated liver disease (ALD) is a common chronic liver disease with increasing incidence worldwide. Alcoholic liver steatosis/steatohepatitis can progress to liver fibrosis/cirrhosis, which can cause predisposition to hepatocellular carcinoma. ALD diagnosis and management are confounded by several challenges. Iron loading is a feature of ALD which can exacerbate alcohol-induced liver injury and promote ALD pathologic progression. Knowledge of the mechanisms that mediate liver iron loading can help identify cellular/molecular targets and thereby aid in designing adjunct diagnostic, prognostic, and therapeutic approaches for ALD. Herein, the cellular mechanisms underlying alcohol-induced liver iron loading are reviewed and how excess iron in patients with ALD can promote liver fibrosis and aggravate disease pathology is discussed. Alcohol-induced increase in hepatic transferrin receptor-1 expression and up-regulation of high iron protein in Kupffer cells (proposed) facilitate iron deposition and retention in the liver. Iron is loaded in both parenchymal and nonparenchymal liver cells. Iron-loaded liver can promote ferroptosis and thereby contribute to ALD pathology. Iron and alcohol can independently elevate oxidative stress. Therefore, a combination of excess iron and alcohol amplifies oxidative stress and accelerates liver injury. Excess iron–stimulated hepatocytes directly or indirectly (through Kupffer cell activation) activate the hepatic stellate cells via secretion of proinflammatory and profibrotic factors. Persistently activated hepatic stellate cells promote liver fibrosis, and thereby facilitate ALD progression.

Alcohol consumption is increasing worldwide, and so is the incidence of alcohol-associated liver disease (ALD).^1^ With no standard laboratory diagnostic test to confirm ALD etiology, asymptomatic early stages, and high costs of disease management, ALD continues to pose challenges on all fronts. Abstinence is the only curative option.^2^

Iron loading is one of the characteristic features of ALD. Even mild to moderate alcohol consumption increases liver iron content.^3^ This can aggravate alcohol-induced liver injury via various mechanisms and promote the pathologic progression of the disease. Knowledge of these mechanisms that mediate liver iron increment in ALD and its consequences at cellular level may help identify cellular/molecular targets and thereby aid in designing better diagnostic, prognostic, and therapeutic approaches for ALD. Such investigations have proved useful in the past. For example, a study showed that liver iron content exhibited a negative correlation with the survival of patients with ALD, and was thus predictive of mortality in patients with alcoholic cirrhosis.^4^

Herein, the cellular mechanisms underlying alcohol-induced liver iron loading are reviewed and how excess iron in patients with ALD can promote liver fibrosis and aggravate disease pathology is discussed.

## High Liver Iron Content in ALD

Patients with ALD/chronic alcohol consumers often show high hepatic iron levels.^5^, ^6^, ^7^, ^8^, ^9^ About 50% of patients with ALD tend to show liver iron excess.^10^ A study showed that the mean liver iron content (measured as μg/100 mg dry weight) in alcoholics was 156.4 ± 7.8, which was significantly higher than that in controls (53 ± 7).^9^ Alcoholic cirrhotic patients frequently show high liver iron content, which is associated with increased mortality.^4^ Increment in liver iron occurs not only because of alcohol consumption but also because of additional factors and mechanisms involving the second hit, such as a high-fat diet in combination with alcohol consumption. Regardless, high liver iron content can contribute to permanent liver injury and hepatocellular carcinoma.^11^ Indeed, with increased serum iron in alcohol consumers, there could also be iron deposition in extrahepatic organs, such as the pancreas and heart, as seen in other iron-loaded conditions.^12^ For example, an autopsy of a 54-year–old woman with ALD showed iron overload in the liver as well as the pancreas, heart, and stomach.^5^

### Pattern of Iron Deposition in Hepatic Cells in ALD

There are two different proposals with regard to iron deposition in the different cell types of the liver. According to one proposal, in mild ALD, iron is preferably deposited in the hepatocytes (parenchymal cells of the liver). As the condition progresses to severe ALD, iron loading is observed more in the Kupffer cells (nonparenchymal cells in liver) compared to hepatocytes.^6^ Pietrangelo^13^ supports the idea of nonparenchymal iron loading in the advanced stages of alcoholic liver fibrogenesis. In contrast, the second proposal suggests that in secondary iron overload syndromes, such as ALD, iron accumulates in the reticuloendothelial system, which includes the Kupffer cells of the liver, and accumulates in the hepatocytes after the reticuloendothelial cells are saturated with iron.^14^ Regardless, in ALD, iron deposition is observed in both hepatocytes and Kupffer cells (ie, in parenchymal and nonparenchymal cells of the liver).

## Cellular Mechanisms that Increase Liver Iron in ALD

Hepcidin, the liver-secreted iron hormone, is the regulator of systemic iron homeostasis.^15^ Alcohol-induced suppression of hepcidin expression is the main cause of systemic iron loading in alcohol consumers. Serum iron loading is further increased by alcohol-induced elevations in the expressions of iron transporters such as duodenal divalent metal-ion transporter 1 (DMT1) and ferroportin in the duodenum. These events enhance intestinal iron absorption (ie, increase iron entry into the circulation),^16^, ^17^, ^18^, ^19^ which forms the basis for liver iron loading in alcohol consumers.

**Figure 1:**
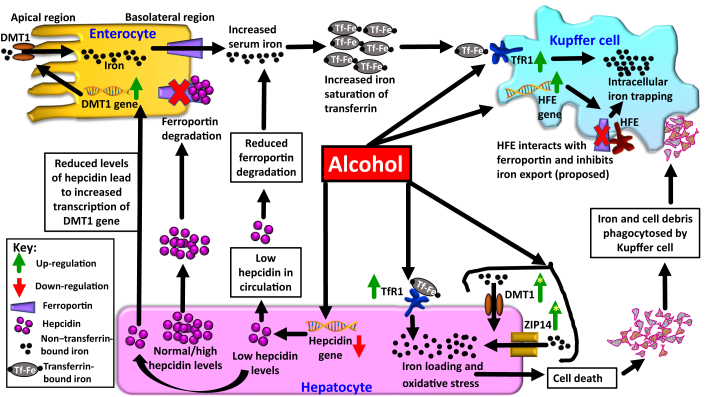
Cellular events underlying alcohol-induced iron loading in different cell types. Alcohol consumption decreases hepcidin levels in the circulation. In turn, this increases intestinal absorption of iron. Elevated serum iron levels cause iron deposition in various cell types, including the hepatocytes and Kupffer cells in the liver, via elevation in transferrin receptor-1 (TfR1) and high iron protein (HFE; proposed). Also, alcohol-induced elevations of non–transferrin-bound iron (NTBI) transporters zinc-regulated, iron-regulated transporter-like protein (ZIP) and divalent metal-ion transporter 1 (DMT1) on hepatocytes, as observed in some studies, aid in hepatocyte iron loading. **Green arrows** with **yellow stars** indicate variability in results with regard to alcohol-induced elevation of these NTBI transporters.

The multiple mechanisms/cellular events that facilitate liver iron loading in ALD are depicted in Figure 1.

### Increased Hepatic TfR1

Increment in hepatic transferrin receptor-1 (TfR1) is one such mechanism that facilitates liver iron loading in ALD. Cellular TfR1 is the receptor for circulating iron-bound transferrin. It facilitates the entry of transferrin-bound iron (TBI) into various cells. Most habitual alcohol consumers/patients with ALD show increased expression of hepatic TfR1 (in hepatocytes), unlike healthy liver tissues.^20^ An increase in the activity of iron regulatory proteins (IRPs) due to alcohol-induced oxidative stress is partly responsible for this increase in TfR1 expression.^6^^,^^21^ Kupffer cells of alcohol-fed rodents have sixfold and ninefold increases in TfR1 gene and protein expressions, respectively.^22^ This collectively indicates that alcohol-induced elevation in TfR1 expression promotes iron uptake in both parenchymal and nonparenchymal cells of the liver (Figure 1). Thus, TfR1 up-regulation may partly explain the liver iron loading in patients with ALD.^20^, ^21^, ^22^ Interestingly, treatment of VL-17A cells with alcohol neither alter the expressions of TfR1 and IRP2 nor alter IRP1 RNA binding activity.^23^ However, a combination of alcohol and iron treatment to rat primary hepatocytes increase the expression of TfR1 (compared with iron alone treatment) partly through the increased activity of IRPs.^23^^,^^24^ On the basis of this, it can be extrapolated that the increased TfR1 expression observed in alcohol consumers is a result of combined effect of alcohol and iron.

Normally, the intracellularly operating IRP–iron response element (IRE) system regulates cellular iron levels by acting on the transcripts for various iron-related genes, including TfR1. Under cellular iron excess, the IRP-IRE system functions to reduce cellular TfR1 to reduce TBI entry into the cells.^25^ Alcohol-induced increment in hepatic TfR1 expression in the presence of hepatic iron loading suggests that alcohol can disturb the aforementioned TfR1-regulatory mechanism and cause or contribute to increased hepatocellular iron uptake.^6^^,^^21^

Macrophages also show iron loading. These cells predominantly acquire iron through phagocytosis of senescent red blood cells. However, these cells express DMT1, TfR1, hemoglobin scavenger receptor (CD163), and natural resistance-associated macrophage protein 1. These proteins are involved in iron uptake and transport,^19^ and may contribute to the increment in liver iron levels.

### Putative Role of HFE Protein

The high iron (HFE) protein may contribute to liver iron accumulation. HFE is a cell surface protein that exhibits multiple functions. First, the HFE can bind to TfR2 to form an iron-sensing complex on the cell membrane. This complex regulates/induces hepcidin expression.^26^ Here, HFE functions as a regulator of hepcidin transcription. Second, HFE can affect the binding of iron-bound transferrin to TfR1. Binding of HFE to TfR1 reduces the affinity of TfR1 to bind to iron-bound transferrin,^27^ thereby reducing cellular iron uptake. Here, HFE functions as a regulator of cellular iron uptake. Third, HFE inhibits cellular iron efflux. Stable transfection-expression of HFE in human colonic carcinoma cell line increases cellular ferritin expression, indicating intracellular iron accumulation/elevation. However, this is independent of transferrin-dependent iron uptake. This suggests that the HFE expression prevents cellular iron efflux and facilitates intracellular iron retention, which results in the aforementioned intracellular ferritin elevation.^28^ Ferroportin is the sole known iron transporter (exporter) on the surfaces of various cell types, including the hepatocytes and Kupffer cells. HFE can interact with ferroportin and inhibit cellular iron release from macrophages (Figure 1).^29^

Alcohol activates *HFE* gene transcription in the Kupffer cells.^22^ Alcohol-exposed rat Kupffer cells show increased Hfe mRNA levels.^19^ On the basis of the postulated function of HFE, this may reduce/inhibit cellular iron export and facilitate iron retention within the Kupffer cells. This may be an additional mechanism causing liver iron loading under the influence of alcohol (Figure 1). Interestingly, duodenal HFE mRNA expression in patients with ALD with iron overload (defined as increased ferritin or transferrin saturation) is significantly higher than in controls, unlike the expression levels in patients with ALD without iron overload and patients with ALD with anemia, in whom levels are similar to controls.^18^ This suggests that the increase in duodenal HFE expression is linked with systemic iron loading, which can subsequently lead to iron deposition in the liver and other organs. On the basis of these data, it appears that HFE function may be cell specific: mediating intracellular iron retention in one cell type, as postulated in case of Kupffer cells, while allowing systemic iron loading through duodenal cells. This hypothesis of the cell-specific nature of HFE needs to be confirmed.

## Enigma Around Ethanol-Induced NTBI Uptake

Depending on the form of iron [TBI or non–transferrin-bound iron (NTBI)], cellular iron uptake can occur via two main mechanisms: TBI uptake and NTBI uptake. NTBI uptake occurs independent of TfR1 and contributes to cell toxicity when in excess. It involves NTBI transporters such as DMT1, zinc-regulated, iron-regulated transporter-like protein 14 (ZIP14) (on hepatocytes), ZIP8, and L-type calcium channels in the cardiomyocytes that are believed to be involved in NTBI uptake.^30^ TBI uptake is regulated by the IRP-IRE system^25^ and functions by down-regulating TfR1 expression under excess iron conditions. In contrast, NTBI uptake occurs despite iron loading. Hepatocytes and parenchymal cells of other tissues, like pancreas and heart, are prone to NTBI uptake. This explains iron loading in the liver and other organs.^30^

ZIP14 and DMT1 can mediate NTBI uptake in hepatocytes (Figure 1). In the context of the effect of alcohol on these NTBI transporters and NTBI uptake, there have been some apparently differing observations. For example, in mice, chronic alcohol and/or iron feeding (15 weeks) caused significantly elevated levels of NTBI in serum and increased the expressions of hepatic DMT1 and ZIP14 at both mRNA and protein levels. This explained the observed increment in their liver iron content^31^ and indicated alcohol-induced elevation in NTBI and in NTBI uptake. In human HepaRG cells (hepatic cell line), ethanol increased total iron content, which appeared to be mediated via elevations in the gene expression of DMT1 and TfR1,^32^ indicating the utility of both NTBI and TBI uptake in the presence of alcohol.

However, in other studies, ethanol exposure dramatically reduced hepatic ZIP14 protein levels in mice,^33^ and there was no major change in hepatic DMT1 in mice after 12 weeks of alcohol feeding.^11^ Because these data are variable, it would be interesting to further investigate and clarify the significance and role of NTBI uptake under the influence of alcohol.

## Alcohol and Liver Ferritin: Some Contradictions

Ferritin (the iron storage protein present intracellularly and in the circulation) is elevated in response to elevation in iron and/or inflammation. It is composed of two types of chains: heavy (H) and light (L). Rats fed with alcohol for 7 weeks showed significantly increased levels of H-ferritin expression in the liver.^34^ Similarly, HepG2 cells treated with alcohol had increased expressions of both H and L ferritin and alcohol increased L-ferritin synthesis in rat hepatocytes.^35^ Alcohol exposure to human hepatoma HepaRG cell line also increased the expression of L-ferritin.^36^ Such an alcohol-induced increase in liver ferritin could be either a rescue mechanism to combat the alcohol-induced elevation in iron levels and store excess iron, or it could be a response to alcohol-induced inflammation or both.

However, a study in mice fed with alcohol for 12 weeks showed decreased hepatic L-ferritin expression, and there were no significant effects at the earlier time points.^11^ Similarly, in VL-17A cells, alcohol did not alter the expression of H-ferritin.^23^ These differential ferritin responses to alcohol require further investigation.

## Combination of Excess Iron and Alcohol Enhances Oxidative Stress and Aggravates ALD Pathology

Under physiological conditions, normal levels of reactive oxygen species (ROS) produced by cellular mechanisms are utilized for cellular purposes, and excess ROS are scavenged/tackled by the endogenous antioxidant mechanisms to prevent ROS-mediated injury. However, excess free iron can accelerate the Fenton reaction, leading to the production of large amounts of ROS that saturate the endogenous antioxidant mechanisms. These free radicals increase oxidative stress and can cause immense cellular and tissue damage^37^ by acting on cellular organelles, DNA, proteins, and lipids.

Both iron overload and alcohol can independently cause oxidative stress and lipid peroxidation. Thus, excess free iron and alcohol act in a synergistic manner to cause liver damage, and the combined effect exacerbates liver injury.^19^ The fibrogenic potential of iron is enhanced when it acts with other hepatotoxins, such as alcohol. The catalytic free iron can directly add to the hepatoxicity of alcohol and/or amplify the generation of cytokines and fibrogenic mediators from the nearby Kupffer cells. Therefore, a slight increase in tissue iron levels in the presence of alcohol (and other metabolites) can accelerate fibrogenesis and advance the liver disease. In the early stages of liver disease, iron-loaded hepatocytes release profibrogenic cytokines and sustain fibrogenesis, whereas at the advanced stages, fibrogenesis is primarily governed by iron-induced hepatocellular necrosis.^13^ Thus, in ALD, excess iron can enhance liver injury by acting as a cofactor for liver fibrogenesis. Also, the combined oxidative stress caused by alcohol and excess iron may cause DNA damage and mutations, resulting in increased predisposition to liver cancer.

## Ferroptosis in Context

### Ferroptosis: An Iron-Dependent Cell Death

Ferroptosis is iron-dependent regulated cell death and is characterized by excessive iron accumulation and lipid peroxidation.^38^ During ferroptosis, glutathione peroxidase is unable to efficiently execute its antioxidant action and repair lipid peroxidation due to the excess of oxidation-reduction–active iron, resulting in unrestricted lipid peroxidation and iron-dependent accumulation of high levels of lipid hydroperoxides.^39^^,^^40^

Ferroptosis is morphologically and biochemically distinct from other cell death patterns such as apoptosis, autophagy, and pyroptosis. Its normal physiological function has not been established yet, but it has a role in pathology. Distinct from its role in hepatocellular carcinoma, where it increases sensitivity to sorafenib (used for liver cancer treatment), in chronic liver diseases, including ALD, ferroptosis aggravates hepatic damage. Generally, it has been implicated in the pathology of liver diseases via several signaling pathways.^38^^,^^39^

### Role of Ferroptosis in ALD Pathology

Alcohol metabolism generates a large amount of acetaldehyde, reduces the levels of the antioxidant glutathione in the mitochondria, and increases ROS production, followed by elevated lipid peroxidation in liver cells. Studies confirm that alcohol treatment induces excessive accumulation of iron in the liver, and increases ROS accompanied by lipid peroxidation, thereby initiating ferroptosis.^41^^,^^42^ The key features of ferroptosis are iron and lipid peroxidation. Both liver iron loading and lipid disorder are features of ALD,^38^ which generates a strong reason for ferroptosis initiation in the livers of patients with ALD.

As previously discussed, excess iron generates free radicals and enhances oxidative stress/injury. The liver is prone to oxidative injury in general. Thus, ferroptosis has a pathogenic role in excess iron–induced hepatic damage and fibrosis, and excess iron is a risk factor for liver fibrosis and cirrhosis.^43^ This explains the role of iron overload in inducing ferroptosis and thereby contributing to ALD pathology.

### Effect of Ferroptosis on Hepatocytes

Long-term alcohol consumption can cause liver iron loading and subsequently promote ferroptosis in the hepatocytes. Hepatocytes have myriads of functions, including regulation of systemic levels of iron, glucose, and lipoproteins. Therefore, regardless of the form of cell death (ferroptosis or otherwise), hepatocyte death or dysfunction is a critical factor for liver injury and failure. Hepatocytes that undergo ferroptosis burst and release damage-associated molecular patterns. These are proinflammatory in nature and activate NOD-like receptor family pyrin domain-containing 3 (NLRP3) inflammasomes in the Kupffer cells, leading to the release of a large volume of proinflammatory cytokines^44^ that aggravate disease pathology.

Thus, excess iron, as found in ALD livers, can induce oxidative stress, cause iron-dependent cell death ferroptosis, promote inflammation, and thereby contribute to liver injury. Unsurprisingly, iron as an initiator of ferroptosis is linked with mortality related to ALD.^41^ Ferroptosis inhibitors, like ferrostatin-1, can rescue the alcohol-induced hepatocyte death and limit alcohol-induced liver injury.^45^ Therefore, ferroptosis appears to be a promising target for ameliorating ALD pathology.

### Cell-Specific Effect of Ferroptosis

Unlike the aforementioned situation, where ferroptosis in hepatocytes exerts a pathologic effect and inhibition of ferroptosis in the hepatocytes is therapeutic, ferroptosis in hepatic stellate cells (HSCs) shows a completely opposite effect. Several studies in animal models have shown that ferroptosis in activated HSCs can reduce liver fibrosis and exert a curative effect. Also, blocking ferroptosis in the HSCs can promote liver fibrosis. Thus, the effect of ferroptosis appears to be cell-type specific. This presents challenges at the therapeutic front because selectively targeting ferroptosis in HSCs can be difficult.^46^ To enable this, specialized systems that exclusively target the HSCs are required.

## Links between Alcohol, Autophagy, Ferritinophagy, and Ferroptosis

### Autophagy: A Cell Survival Mechanism that Can also Promote Cell Death

Autophagy is a conserved catabolic cellular process triggered following an insult or stress. It degrades damaged organelles and extra/unnecessary proteins, aiming to maintain a balance between protein degradation, synthesis, and recycling of cellular components. It involves the formation of vesicles called autophagosomes, which deliver the cytosolic cargo to lysosomes for degradation, and recycling it back to the cytosol. Dysregulation of autophagy has been implicated in metabolic and neurodegenerative diseases, inflammation, aging, and cancer. In the liver, autophagy maintains the cellular functionality of hepatocytes.^47^^,^^48^

### Autophagy Degrades Ferritin

**Figure 2:**
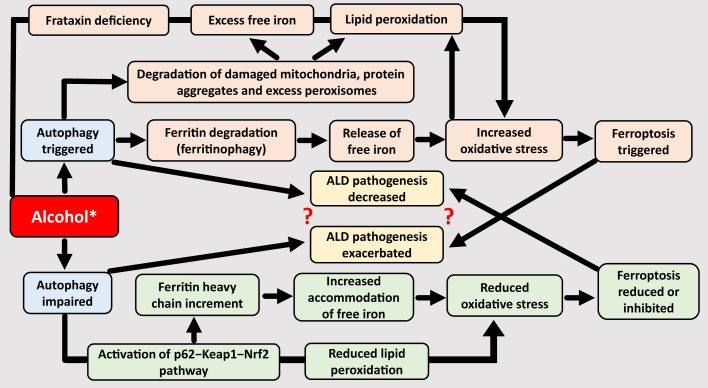
Putative associations between autophagy, ferritinophagy, and ferroptosis in the presence of alcohol. Alcohol has a dual effect of autophagy (ie, it can both stimulate and impair autophagy). This differential effect of alcohol on autophagy and the consequent ambiguity is indicated through an **asterisk** in the figure. Degradation of ferritin via autophagy is ferritinophagy. Ferritinophagy can trigger ferroptosis, whereas increment in ferritin can increase the probability of accommodating free iron, thereby reducing excess iron–induced oxidative stress, and consequently reducing ferroptosis. Autophagy can also trigger ferroptosis through ferritinophagy-independent routes, such as those involving frataxin deficiency, and degradation of damaged or excess cellular components that eventually increases free iron levels and/or lipid peroxidation.^49^^,^^50^ Interestingly, although autophagy can trigger ferroptosis, which can exacerbate alcohol-associated liver disease (ALD) pathology, autophagy appears to also impart a protective effect and decrease or blunt ALD pathology. These apparently opposing concepts have been indicated by question marks in the figure and need further clarification.

Autophagy degrades ferritin, the iron-storage protein. This is called ferritinophagy. Ferritin degradation inside the autolysosomes leads to the release of iron from ferritin. This released free iron is likely to be transported back to cytosol, leading to increment in ROS and oxidative stress, which can trigger ferroptosis. Thus, ferritinophagy can play a role in triggering ferroptosis (Figure 2),^49^, ^50^, ^51^, ^52^, ^53^ and ferritin negatively regulates ferroptosis.^54^ In HepG2 cells, autophagy inhibition increased ferritin heavy chain production.^55^ In theory, this could aid in scavenging/accommodating free iron within ferritin, leading to reduction in oxidative stress and, thereby, reduction in ferroptosis. Collectively, data suggest that ferritinophagy can promote ALD pathology, in part via ferroptosis, because ferroptosis aggravates liver pathology (Figure 2).

### Autophagy Shows Divergent Relation with ALD: Further Clarity Needed

There are differing data on the effect of alcohol on autophagy. Studies indicate that alcohol exposure can increase autophagosome formation and trigger autophagy. This is a protective mechanism that selectively removes damaged mitochondria and hepatic lipids. However, alcohol can also impair lysosome function or lysosomal biogenesis, leading to deficient autophagy in the hepatocytes, and contribute to ALD pathology (Figure 2).^56^ These apparently contrasting effects could be due to differential effects of acute and chronic alcohol on autophagy, due to differential effects of alcohol itself on autophagy, or the role of autophagy in both cell survival and cell death; the latter depending on cell type and context.^57^ The reason(s) for these differential effects need to be identified.

There are conflicting inferences involving autophagy, ferroptosis, and ALD pathology (Figure 2). Inhibition of autophagy in alcohol-fed mice increases hepatoxicity, steatosis, oxidative stress, and hepatocyte apoptosis, and activation of autophagy blunts the alcohol-induced steatosis.^56^ This indicates a protective role of autophagy under alcoholic conditions. However, experiments in HepG2 cells show that inhibition/impairment of autophagy activates the p62-Keap1-Nrf2 pathway. This is protective against alcohol-induced ferroptosis,^55^ and thereby should reduce/decelerate ALD pathology. Unlike the previous case, this presents autophagy impairment as having a protective role under alcoholic conditions (Figure 2).

These conflicting relationships, which infer that autophagy can trigger ferroptosis but also decrease ALD pathology, and impaired autophagy can reduce ferroptosis but also accelerate ALD pathology, require further clarification.

## Intercellular Events Underlying Iron-Aggravated Liver Fibrosis in ALD

Iron loading is one of the independent risk factors for fibrosis in ALD.^58^ Thus, it is important to review the intercellular events involved in the iron-facilitated progression to liver fibrosis.

**Figure 3:**
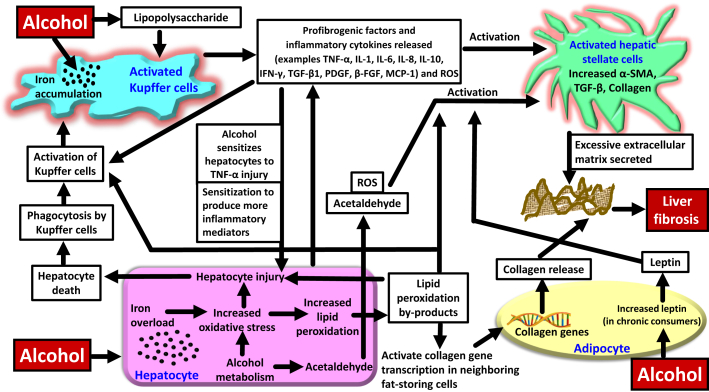
Intercellular events depicting the role of iron in enhancing alcohol-induced liver fibrosis. Alcohol can cause iron loading in the hepatocytes and Kupffer cells. Oxidative injury to hepatocytes due to excess iron and alcohol can lead to hepatocyte death. Kupffer cells phagocytose dead/damaged hepatocytes and get activated. Activated Kupffer cells release profibrotic cytokines and activate the hepatic stellate cells (HSCs). In addition, profibrotic/inflammatory cytokines released from injured hepatocytes together with reactive oxygen species (ROS) and acetaldehyde produced from alcohol metabolism in the hepatocytes activate the HSCs. Following activation, HSCs secrete profibrotic factors and excessive extracellular matrix that collectively form the basis for liver fibrosis. Adipocytes also play a role in promoting alcohol-induced liver fibrosis, and together with excess iron, the pathology may be aggravated. β-FGF, β-fibroblast growth factor; IFN-γ, interferon-γ; MCP-1, monocyte chemoattractant protein-1; PDGF, platelet-derived growth factor; α-SMA, α-smooth muscle actin; TGF, transforming growth factor; TNF-α, tumor necrosis factor-α.

Figure 3 summarizes the intercellular interactions, and the ways in which iron loading can exacerbate liver injury in ALD and promote liver fibrosis. Table 1^59^, ^60^, ^61^, ^62^, ^63^, ^64^, ^65^, ^66^, ^67^, ^68^, ^69^, ^70^, ^71^, ^72^, ^73^, ^74^, ^75^ presents an overview of the effect of iron overload on some of the core cell types in the liver. Each cell type of the hepatic lobule is actively involved in the fibrogenic process. The main cell types involved in this process are the hepatocytes, Kupffer cells, and HSCs, whereas the liver endothelial cells (Table 1). Fat-storing cells (described in the subsequent section) also play a role.

**Table 1:** Overview of the Most Prominent Effects of Iron Overload on the Core Liver Cells and the Associated Underlying Mechanisms

Liver cell type and its generic function	Prominent effects of iron overload	Underlying cellular mechanisms in context of iron loading
Hepatocytes (hepatic parenchymal cells, make majority of liver parenchyma and exhibit various functions, including sensing iron in the circulation and secreting the iron-regulating hormone hepcidin)^15^	Increased oxidative stress, resulting in damage to cellular organelles, lipids, proteins, and DNA^44^^,^^59^	Excess iron–induced elevation in ROS production is via the Fenton reaction^44^^,^^59^
	Cell death	Excess ROS causes lipid peroxidation, which contributes to different types of cell deaths, including ferroptosis.^59^, ^60^, ^61^
	Increased synthesis and secretion of hepcidin^15^	Hepcidin is induced via the BMP-SMAD pathway.^15^ (Notably in ALD, hepcidin synthesis and secretion is reduced due to alcohol-induced inhibition of the BMP-SMAD pathway,^62^ attenuation of JAK/STAT signaling,^16^ and oxidative stress.^19^^,^^44^)
Kupffer cells (hepatic nonparenchymal cells, clear microorganisms, dead cells, debris, and circulating endotoxin)^63^	Increased production of inflammatory cytokines^64^	Iron loading can activate NF-κB,^44^^,^^65^ which can stimulate the production of proinflammatory cytokines, like TNF-α and IL-6.^66^
	Enhanced inflammatory response to LPS^22^^,^^67^	Disruption of mitochondrial homeostasis and increased generation of mitochondrial superoxide partly promote inflammatory response to LPS.^67^
HSCs (hepatic nonparenchymal cells, generally quiescent, mediate wound healing following an injury)	Persistent cell activation and proliferation, leading to promotion of fibrosis^59^	Stimulation of the expressions of type I collagen and α-SMA (makers of fibrosis), increased production of TGF-β1, and activation of TGF-β pathway.^68^^,^^69^
	Extracellular ferritin stimulates inflammatory pathway in HSCs^70^	Activated HSCs exhibit a receptor for H-ferritin. Binding of ferritin (H-ferritin) activates NF-κB through PI3 kinase, PKCζ, MEK1/2, MAPK, and IKKα/β. Thereby, extracellular ferritin acts as a proinflammatory mediator.^70^
LSECs (hepatic nonparenchymal cells, form a fenestrated endothelium that allows movement of selective molecules, and play a role in clearance of macromolecules from blood,^63^ differentiated LSECs maintain HSC quiescence and help prevent fibrosis^71^)	Induce hepcidin production in the hepatocytes^72^	LSECs can sense iron and produce BMPs in response. BMPs 2 and 6 can induce hepcidin synthesis in hepatocytes via BMP-SMAD pathway.^15^^,^^72^ (Note that in ALD, hepcidin synthesis and secretion is reduced due to the previously explained reasons.)
	Following chronic liver injury (including persistent iron overload), LSECs can dedifferentiate and activate the HSCs, which leads to increased production of extracellular matrix, LSECs lose their fenestrations (defenestration) and function^63^^,^^71^^,^^73^	The effect of iron on LSEC defenestration is not direct. Iron-stimulated hepatocytes secrete nerve growth factor. This acts on nerve growth factor receptor on LSECs and triggers defenestration (in part).^73^ Also, excess iron–induced mitochondrial oxidative damage activates transcription factor Nrf2 in LSECs.^74^ Continuous activation of Nrf2 inhibits autophagy.^71^ Normally, autophagy helps maintain LSEC phenotype (ie, fenestrae by controlling nitric oxide bioavailability).^75^ Thus, iron overload can cause LSEC defenestration over time.^71^

ALD, alcohol-associated liver disease; BMP, bone morphogenetic protein; HSC, hepatic stellate cell; IKK, inhibitory kappa B kinase; JAK/STAT, Janus kinase/signal transducer and activator of transcription; LPS, lipopolysaccharide; LSEC, liver sinusoidal endothelial cell; MAPK, mitogen-activated protein kinase; MEK, mitogen activated protein kinase extracellular signal-regulated kinase; Nrf2, nuclear factor erythroid 2-related factor 2; PI3, phosphatidylinositol 3-kinase; PKC, protein kinase C; ROS, reactive oxygen species; α-SMA, α-smooth muscle actin; TGF, transforming growth factor; TNF-α, tumor necrosis factor-α.

### Interaction between Hepatic Stellate Cells, Hepatocytes, and Kupffer Cells

The HSCs play a crucial role in liver fibrogenesis. Activation of HSCs is a normal phenomenon that mediates wound repair. Following repair, HSCs either revert to their quiescent state or undergo apoptosis. However, persistent liver insults keep the HSCs continuously activated. These HSCs secrete excessive amounts of profibrogenic factors and extracellular matrix that collectively induce a pathologic state and form the basis of liver fibrosis. When liver iron exceeds 60 μmol/g, the HSCs get activated. Iron-induced promotion of fibrogenic mechanisms has been shown in murine HSCs, and the contribution of excess iron in enhancing liver fibrosis is well established.^59^^,^^68^^,^^76^

Iron-loaded hepatocytes release profibrogenic factors and can directly activate the HSCs (Figure 3). In addition, these hepatocytes can release profibrotic/proinflammatory factors and stimulate the Kupffer cells.^77^ Alcohol increases the translocation of lipopolysaccharide from the intestine to the liver, which additionally stimulates the Kupffer cells. Once activated, the Kupffer cells release proinflammatory and profibrotic factors, such as tumor necrosis factor (TNF)-α, IL-1, IL-6, IL-8, IL-10, interferon-γ, transforming growth factor-β1, platelet-derived growth factor, β-fibroblast growth factor, monocyte chemoattractant protein-1, and ROS. These cytokines, in turn, activate the HSCs (Figure 3).^77^, ^78^, ^79^, ^80^ Injured hepatocytes can activate the HSCs directly, or indirectly by stimulating the Kupffer cells to secrete profibrotic factors which, in turn, activate HSCs. Regardless, on activation, HSCs differentiate into myofibroblasts and synthesize and release excessive amounts of extracellular matrix composed of elastin, collagen, and other matrix proteins, thereby exhibiting liver fibrosis (Figure 3).^59^^,^^77^

Activation of NF-κB correlates with liver inflammation and fibrosis in ALD.^81^ Alcohol-induced accumulation of iron in Kupffer cells can activate NF-κB and worsen experimental ALD/alcoholic steatohepatitis.^22^^,^^65^^,^^82^ Alcoholics show increased levels of lipopolysaccharide in the circulation. Iron and lipopolysaccharide are believed to activate NF-κB in the Kupffer cells and induce the synthesis of proinflammatory cytokines, like TNF-α.^19^ TNF-α plays an important role in liver injury. Normally, hepatocytes are not negatively affected by TNF-α. However, alcohol sensitizes the hepatocytes to injury by TNF-α and causes hepatocyte cell death via apoptosis.^80^^,^^83^ These dead cells are engulfed by the Kupffer cells (Figure 3). In animal models, Kupffer cell depletion or inactivation dampens alcohol-induced effects, such as inflammation, fatty liver, and necrosis. Thus, Kupffer cells play an important role in the pathologic progression of ALD.^19^

### The Role of Adipocytes

In addition to Kupffer cells and HSCs, surrounding cells such as the adipocytes from adipose tissue, are involved in ALD pathogenesis. Independent of the effect of alcohol, lipid peroxidation by-products released from iron-overloaded hepatocytes are able to stimulate collagen gene transcription in the neighboring fat-storing cells directly or via activation of Kupffer cells.^84^ This may further aggravate ALD pathogenesis in cases with iron overload. Notably, excess iron–generated ROS and lipid peroxidation by-products can activate both Kupffer cells and HSCs (Figure 3).^13^

Alcohol induces inflammation in the adipose tissue. Alcohol-induced lipolysis in the adipocytes (which promotes hepatic steatosis) together with inflammatory responses in the macrophages release increased levels of free fatty acids, adipokines (such as leptin), and cytokines (such as TNF-α and IL-6) into the portal circulation.^85^^,^^86^ These adipokines, like leptin, have proinflammatory effects on the liver. Leptin (along with other endocrine factors) activates the HSCs and Kupffer cells (that produce increased TNF-α), and thereby promotes hepatic inflammation and fibrosis (Figure 3). High levels of TNF-α can damage the liver hepatocytes, as discussed previously.^86^ Also, leptin and acetaldehyde together can enhance the production of IL-6 in the HSCs (Figure 3).^87^ Leptin levels correlate with liver disease severity in patients with alcoholic cirrhosis.^85^ In addition, iron loading in the adipocytes reduces the production of the anti-inflammatory adipokine adiponectin. This can further promote inflammation and contribute to liver injury.^88^

Although the aforementioned cellular interactions showcase an iron perspective, ALD pathology is additionally driven by both adaptive and innate immune systems and involves the recruitment of various immune cells that generate a proinflammatory environment in the liver.^89^ As such, the liver has abundant lymphocytes scattered through its parenchyma, and it is also rich in cells of the innate immune system, such as the natural killer cells.^63^ Iron plays a role in liver pathology via the immune cells. For example, iron deficiency dampened concanavalin A–induced intrahepatic inflammation in mice. It also reduced intrahepatic lymphocyte infiltration.^90^

## Low Liver Iron Content: A Phenomenon to Be Investigated

In a study by Varghese et al,^11^ mice models showed gradual elevation of serum iron levels during 12 weeks of alcohol feeding. Elevations in duodenal ferroportin (gradually increased at 8 weeks and further at 12 weeks) and duodenal DMT1 (significantly increased at 8 weeks but decreased to control levels at 12 weeks) supported this increment in serum iron. In contrast to these elevations, hepatic and serum hepcidin expression gradually decreased during the 12 weeks of alcohol exposure.^11^ This alcohol-induced decrement in hepcidin is an expected response and is also seen in patients with ALD.^16^, ^17^, ^18^, ^19^ Herein, the lack of hepcidin up-regulation despite elevation in serum iron levels reiterates the insensitivity of hepcidin to increasing systemic iron levels in the presence of alcohol.

Unlike the frequently observed hepatic iron elevation in alcoholics, hepatic iron levels in mice models decrease after 12 weeks of alcohol feeding.^11^ The pattern of liver iron decrement matches fully with the patterns of decrements of hepatic TfR1 and hepatic ferritin expressions through the 12 weeks of alcohol exposure. This decrease in liver iron content is an unexpected response because several studies in humans have shown increased liver iron content in alcohol consumers/patients with ALD.^5^, ^6^, ^7^

Varghese et al^11^ attributed the decrement in hepcidin expression partly to decreased hepatic iron levels. The authors proposed that this could be due to alcohol-induced hepatomegaly and alcoholic steatosis and/or mobilization of iron to other tissues. The idea of mobilization of iron from liver to other tissues was supported by their observation that hepatic ferroportin expression showed a tendency to increase after 4 and 12 weeks of alcohol exposure, which would facilitate cellular iron egress.^11^ The reason for decrement in liver iron content needs to be fully understood, particularly because it involves the function of ferroportin, the sole known unidirectional cellular iron transporter.

## Liver Iron Loading in ALD: Diagnostic, Prognostic, and Therapeutic Perspectives

### Liver Iron and ALD Diagnosis and Prognosis

Currently, there is no single diagnostic test to confirm ALD.^91^ One of the challenges for diagnosis is that the symptoms of ALD are not obvious in the early stages. Suspected cases are often tackled based on patient-derived information about their alcohol intake (patient history) supported by laboratory tests. Crabb et al^92^ have reviewed this topic in detail. Liver iron loading by itself cannot be used for the diagnosis of ALD or any chronic liver disease because there are several other liver conditions, such as hemochromatosis and nonalcoholic fatty liver disease, that show high liver iron content.^59^ An old study indicated that liver iron in ALD has a prognostic value. It showed that patients with alcoholic cirrhosis with detectable liver iron had a lower survival rate than those without.^4^ However, other studies suggest that hepatic iron overload is a poor prognostic factor in ALD.^93^

### Liver Iron and ALD Therapeutics: Alcohol Abstinence

Although there are US Food and Drug Administration–approved therapies for alcohol use disorders that help reduce cravings for alcohol,^92^^,^^94^ there is no US Food and Drug Administration–approved drug to treat ALD.^95^ Alcohol abstinence is the only curative option, and liver transplantation is the definitive treatment for liver diseases (including ALD) in the end stage.

Cessation of alcohol has shown to reduce liver iron deposits. For example, patients with ALD who abstained for >3 months had reduced liver iron content compared with patients with ALD with active alcoholism (average intake of 164.4 g/day).^96^ Also, drinking lesser amount of alcohol has shown to cause lesser liver iron deposition. For example, in a study, mean liver iron concentrations were significantly higher in alcoholic patients (who drank >80 g/day for ≥3 years before and inclusive of the study period) compared with controls who did not drink excessive amounts of alcohol (ie, did not drink >20 g/day).^9^

### Liver Iron and ALD Therapeutics: Discussing Phlebotomy

Hemochromatosis is an iron-overload disease in which patients show high systemic and liver iron loading, in addition to iron deposition in other organs.^30^ For patients with hemochromatosis who show high iron loading, life-long periodic phlebotomy is the standard of care, which reduces the level of iron, thereby limiting excess iron–induced organ damage. In a patient with ferroprotein disease (hereditary iron loading disorder), long-term phlebotomy decreased hepatic iron accumulation.^97^

This questions whether phlebotomy could be used for patients with ALD who show liver iron overload. First, just like in case of hemochromatosis, where not all patients demonstrate enough iron overload to cause organ damage,^30^^,^^98^ not all patients with ALD show liver iron loading.^10^^,^^11^^,^^99^ Some patients with ALD may be anemic.^100^ Second, in patients with ALD who show liver iron loading, the levels hardly ever surpass two to three times the upper limit of the norm.^101^ Third, phlebotomy has several limitations, one of which is the possibility of developing anemia.^99^ Therefore, although phlebotomy is a suitable option for iron-overloaded patients with hemochromatosis, it is not a suitable therapeutic option for patients with ALD.

### Liver Iron and ALD Therapeutics: Iron Chelation

In general, apart from phlebotomy, another therapeutic approach for reducing liver iron content is iron chelation by using chelators like deferoxamine, deferiprone, and deferasirox.^102^, ^103^, ^104^ Deferiprone decreases hepatocyte iron overload in chronically ethanol-fed rats.^105^ A novel iron chelator, M30, reduces alcohol-indued injury in rat hepatocytes and attenuates ethanol-induced apoptosis, oxidative stress, and secretion of inflammatory cytokines.^106^ Thus, these chelators are potential therapeutics for ALD cases that show liver iron overload.

Naturally occurring compounds (namely, flavonoids) are also potential therapeutic agents. These impair ALD pathologic progression by maintaining iron balance. For example, quercetin, which exhibits iron-chelating and antioxidant properties, dampens alcohol-induced liver damage in mice.^44^ Such natural compounds can be tested in alcohol-treated animal models and subsequently relevant clinical trials can be established.

### Liver Iron and ALD Therapeutics: Synthetic Hepcidin

Hepcidin deficiency is the main cause of iron loading in patients with ALD.^16^^,^^23^ Therefore, hepcidin treatment is a promising therapeutic approach. Natural hepcidin is expensive and has undesirable pharmacologic properties, such as having a short half-life. In contrast, minihepcidins are synthetic in nature. These mimic the action of hepcidin and are pharmacologically more favorable.^99^ I.P. injections of minihepcidin in mice models of hemochromatosis show significant reductions in liver iron loading.^107^ Similar studies in alcohol-fed animal models can be used to extrapolate whether this approach would be successful in reducing liver iron loading in patients with ALD.

### Liver Iron and ALD Therapeutics: Targeting Ferroptosis

Interestingly, not the liver iron loading itself, but ferroptosis, the iron-dependent process that contributes to liver damage in ALD, has been targeted for therapy. Ferroptosis inhibitors and repressors have shown protective effects against alcohol-induced liver damage. For example, the ferroptosis inhibitor ferrostatin-1 reduced lipid peroxidation and alcohol-induced liver injury *in vivo*.^41^ Another ferroptosis inhibitor, dimethyl fumarate, significantly improved alcohol-induced liver injury in ethanol-fed mice.^108^ Also, deficiency of intestinal sirtuin 1 (SIRT1) in mice has shown protection from alcohol-induced hepatic injury via mitigation of ferroptosis.^42^

Frataxin is a mitochondrial protein that predominantly participates in iron homeostasis and oxidative stress. A study showed that alcohol reduced the expression of frataxin, and the deficiency of frataxin increased sensitivity to alcohol-induced ferroptosis (Figure 2). Restoration of frataxin reversed this effect.^109^ Thus, frataxin can be an additional therapeutic target to tackle ALD.

## Summary

Increased serum iron due to chronic alcohol consumption increases iron uptake in the hepatocytes and Kupffer cells, facilitating both parenchymal and nonparenchymal iron loading in the liver, and in parenchymal cells of other organs. Hepatic iron deposition is mediated via up-regulation of TfR1 and HFE (proposed). Both iron and alcohol can independently induce oxidative stress, so the combined effect accelerates hepatic injury. Excess iron–stimulated hepatocytes and Kupffer cells secrete inflammatory and profibrogenic factors that activate the hepatic stellate cells. Chronic activation of hepatic stellate cells mediates the development of liver fibrosis. Iron loading promotes ALD progression via induction of oxidative stress and the activation of HSCs and Kupffer cells. Other cells, such as the liver sinusoidal endothelial cells, the liver immune cells (from both adaptive and innate immune systems), and the adipocytes, also contribute to the iron-mediated liver injury in ALD.
